# Anatomy and Function of Ventral Tegmental Area Glutamate Neurons

**DOI:** 10.3389/fncir.2022.867053

**Published:** 2022-05-20

**Authors:** Jing Cai, Qingchun Tong

**Affiliations:** ^1^Brown Foundation Institute of Molecular Medicine for the Prevention of Human Diseases, UTHealth McGovern Medical School, Houston, TX, United States; ^2^Neuroscience Program, The University of Texas MD Anderson Cancer Center UTHealth Graduate School of Biomedical Sciences, Houston, TX, United States

**Keywords:** VTA, VGLUT2, dopamine, neural circuits, reward, addiction

## Abstract

The ventral tegmental area (VTA) is well known for regulating reward consumption, learning, memory, and addiction behaviors through mediating dopamine (DA) release in downstream regions. Other than DA neurons, the VTA is known to be heterogeneous and contains other types of neurons, including glutamate neurons. In contrast to the well-studied and established functions of DA neurons, the role of VTA glutamate neurons is understudied, presumably due to their relatively small quantity and a lack of effective means to study them. Yet, emerging studies have begun to reveal the importance of glutamate release from VTA neurons in regulating diverse behavioral repertoire through a complex intra-VTA and long-range neuronal network. In this review, we summarize the features of VTA glutamate neurons from three perspectives, namely, cellular properties, neural connections, and behavioral functions. Delineation of VTA glutamatergic pathways and their interactions with VTA DA neurons in regulating behaviors may reveal previously unappreciated functions of the VTA in other physiological processes.

## Introduction

The ventral tegmental area (VTA) located in the midbrain controls diverse behavioral repertoire, including reward processing, aversion, stress modulation, drug addiction, learning, and memory (Haber and Fudge, 1997; Ikemoto, 2007; Arias-Carrion et al., 2010; Polter and Kauer, 2014). The VTA functional diversity is partly reflected by its cellular and circuit heterogeneities. The VTA is composed of ∼60% dopaminergic neurons (DA neurons), ∼35% GABAergic neurons (GABA neurons), and ∼5% glutamate neurons (glutamate neurons) (Nair-Roberts et al., 2008; Yamaguchi et al., 2015). Other than these three classical neurotransmitters, VTA neurons also release peptides, including cholecystokinin, neurotensin, corticotropin-releasing factor, brain-derived neurotrophic factor, and calbindin (Seroogy et al., 1988; Jayaraman et al., 1990; Hyman et al., 1994; Liang et al., 1996; Grieder et al., 2014). Co-release of classical neurotransmitters and neuropeptides increases the cellular heterogeneity of VTA neurons. At the circuit level, these diverse VTA neurons make direct synaptic contacts with multiple brain regions, including the prefrontal cortex (PFC), the nucleus accumbens (NACc), the pedunculopontine tegmentum (PPTg), the laterodorsal tegmentum nucleus (LDTg), the lateral habenula (LHb), the periaqueductal gray (PAG), the bed nucleus of the stria terminalis (BNST), the lateral hypothalamus (LH), the ventral pallidum (VP), and the dorsal raphe nucleus (DRN) (Morales and Margolis, 2017). Intricate interactions among different VTA neuron populations and their diverse input and output projections mediate the behavioral repertoire of VTA function.

The DA neurons in the VTA have been a hot topic and are considered the major therapeutic target for treating reward-related disorders, such as drug addictions and mood disorders due to their key roles in directing reward-related responses (Price and Drevets, 2010; Polter and Kauer, 2014). Other than VTA DA neurons, increasing studies suggest that VTA GABA neurons are also important for behavioral regulation by forming local synapses onto DA neurons or sending projections to remote brain sites. VTA GABA neurons have been found to modulate reward consumption, depression, stress, and sleep by altering DA release from neighboring DA neurons (Van Zessen et al., 2012; Bouarab et al., 2019; Chowdhury et al., 2019; Yu et al., 2019, 2021; Galaj et al., 2020). Long-range connections of GABA neurons also intertwine with VTA dopaminergic circuits (Beier et al., 2019; Bouarab et al., 2019), suggesting that the function of VTA GABA neurons is at least partially dependent on DA release. Relatively less has been focused on VTA glutamate signaling due to their rare existence in the VTA. However, recent studies suggest that VTA glutamate neurons regulate reward reinforcement, aversive behaviors, wakefulness, and defensive behaviors (Mangieri et al., 2019; Yu et al., 2019; Barbano et al., 2020; Zell et al., 2020), emphasizing the importance of VTA glutamate release. This review aims to give a general overview of VTA glutamate neurons in the aspects of cellular features, neural connections, and behavioral functions that can be DA dependent or independent.

## Molecular and Electrophysiological Features of Ventral Tegmental Area Glutamate Neurons

In the brain, glutamate is synthesized from glutamine by glutaminase and then packaged into vesicles by vesicular glutamate transporters (Vgluts) for its synaptic release (Takamori et al., 2000). Glutamate neurons in the VTA mainly express Vglut2 but not Vglut1 or Vglut3 (Yamaguchi et al., 2007, 2011). Vglut2-expressing glutamate neurons are mostly located in the anterior and middle line of the VTA, where these neurons outnumber DA neurons (Yamaguchi et al., 2007). In addition, Vglut2-expressing neurons form asymmetry synapses and release glutamate to downstream targets once activated, confirming that VTA Vglut2-expressing neurons release glutamate and form excitatory synapse (Chuhma et al., 2004; Hnasko et al., 2012). In addition, about 35% of NACc-projecting VTA neurons and 66% of PFC-projecting VTA neurons contain Vglut2 (Stuber et al., 2010; Tecuapetla et al., 2010; Yamaguchi et al., 2011; Gorelova et al., 2012), implicating a role for glutamate release from the VTA to both NACc and PFC in mediating the VTA function.

The VTA glutamate neurons are heterogeneous. A subset of these neurons only releases glutamate, while others also express tyrosine hydrolase (TH) and may co-release DA (Hnasko et al., 2010, 2012; Stuber et al., 2010; Tecuapetla et al., 2010; Morales and Root, 2014; Root et al., 2016). Surprisingly, about 50% of Vglut2^+^ TH^+^ expressing neurons do not express vesicular monoamine transporter 2 (Vmat2), DA transporter (DAT), or DA receptor 2 (D2) (Li et al., 2013), which are either required for DA release or normally expressed in VTA DA neurons, indicating that this subset of Vglut2^+^ TH^+^ expressing neurons may solely release glutamate despite expressing TH. The release of glutamate from VTA DA neurons is speculated to be responsible for fast responses (Chuhma et al., 2004; Lavin et al., 2005). Interestingly, there appears to be a development requirement for Vglut2 expression in the VTA. The knockout of Vglut2 from midbrain DA neurons in the neonatal stage results in morphological abnormalities of these neurons (Fortin et al., 2012; Papathanou et al., 2018) and alterations in risk-taking behaviors as well as DA responses to amphetamine (Birgner et al., 2010; Alsio et al., 2011; Berube-Carriere et al., 2012). However, the knockout of Vglut2 from DA neurons in the adult stage had minimal effects on either morphology or behaviors (Papathanou et al., 2018). These contrasting observations indicate an important function of developmentally transient Vglut2 expression in VTA DA neurons. The transient expression of Vglut2 during development in a subset of VTA-DA neurons is supported by a reduced proportion of VTA Vglut2^+^ TH^+^ neurons in adulthood compared to neonates (Berube-Carriere et al., 2009, 2012; Moss et al., 2011). In line with this, neonatal knockout of Vmat2 from Vglut2 expressing neurons also leads to almost complete deletion of Vmat2 expression in the VTA and results in developmental abnormalities and death (Cai et al., 2021), reminiscent phenotypes of knockout of Vmat2 at the whole body (Wang et al., 1997), suggesting extensive transient co-localization between Vmat2 and Vglut2 in the VTA during development. It is interesting to note that the off-expression of Vglut2 in adulthood is an integral part of normal VTA DA neuron development and function as over-expression of Vglut2 in these neurons in adulthood causes neuronal death (Steinkellner et al., 2018). These observations suggest an importance of developmental Vglut2 expression, potentially *via* glutamate release, in the normal development of VTA DA neurons. Thus, it appears that VTA Vglut2^+^ TH^+^ neurons in adulthood may represent only a small subset of Vglut2^+^ TH^+^ neurons in neonates, in which residual Vglut2 expression remains.

A small subset of VTA glutamate neurons co-expresses vesicular GABA transporter (Vgat) and co-releases GABA (Root et al., 2014; Yoo et al., 2016). Most VTA Vglut2^+^Vgat^+^ neurons are located in the anterior portion of the interfascicular nucleus (IF) (Root et al., 2020). These neurons can establish adjacent asymmetric and symmetric synapses on downstream neurons (Root et al., 2014), suggesting an intriguing co-existence of both excitatory glutamatergic and inhibitory GABAergic synapses formed by these individual presynaptic neurons. Consistently, activation of these neurons is able to induce both fast excitation and inhibition in downstream neurons, the net responses of which seem to be projection-specific (Root et al., 2014; Yoo et al., 2016). Another small subset of Vglut2^+^ neurons expresses both TH and glutamic acid decarboxylases (GADs) (Root et al., 2014), another marker for GABAergic neurons. However, whether these neurons co-release all three neurotransmitters remains to be demonstrated. Although VTA neurons are known to co-express abundant neuropeptides (Seroogy et al., 1988; Jayaraman et al., 1990; Hyman et al., 1994; Liang et al., 1996; Grieder et al., 2014), relatively less is known about whether VTA glutamate neurons also co-release peptides (Wise and Morales, 2010).

The VTA glutamate neurons exhibit a unique electric property. The hyperpolarization-activated cation current (*I*_h_) mediated by hyperpolarization-activated cyclic nucleotide-gated channels is an important contributor to both resting membrane potential and dendritic integration (Pape, 1996; Robinson and Siegelbaum, 2003). Similar to medial, but contrary to lateral VTA DA neurons, VTA glutamate neurons have a small or no *I*_h_, which leads to a lower resting membrane potential (Hnasko et al., 2012). In addition, compared to lateral DA neurons, VTA glutamate neurons also present a shallower afterhyperpolarization (AHP) and consequent higher firing rate (Hnasko et al., 2012). In addition, *ex vivo* electrophysiological recordings reveal that VTA glutamate neurons present a relatively more hyperpolarized resting membrane potential, greater rheobase, and lower spontaneous firing frequency compared to GABA neurons (Miranda-Barrientos et al., 2021). These studies suggest that VTA glutamate neurons are more excitable than other VTA neurons.

## Neural Connections of Ventral Tegmental Area Glutamate Neurons

### Intrinsic Connections

Accumulating data support an existence of strong intrinsic connections among VTA neurons. Anatomical and electrophysiological data reveal that VTA GABA neurons form local synapses onto other VTA neurons (Omelchenko and Sesack, 2009; Matsui and Williams, 2011). For DA neurons, the inhibitory input from local GABA neurons appears to be weaker than long-range inhibitory ones (Omelchenko and Sesack, 2009). However, disrupting local VTA GABA release causes major malfunctions in stress and anxiety modulation, which can be rescued by restoring GABAergic control on DA neurons (Van Zessen et al., 2012; Bouarab et al., 2019; Chowdhury et al., 2019; Yu et al., 2021), suggesting the importance of intrinsic GABAergic action in controlling VTA DA neurons and maintaining normal behavioral responses.

Ultrastructural studies suggest that ∼50% of VTA fibers forming local synaptic contacts contain Vglut2, and these glutamatergic fibers establish asymmetric synapses on both DA and non-DA neurons (Dobi et al., 2010). Optical stimulation of VTA glutamate neurons elicits AMPA/NMDA receptor-dependent firing in NACc-projecting DA neurons *via* monosynaptic connections, suggesting a local excitatory synapse connection between glutamate and DA neurons (Wang et al., 2015). This local excitatory connection is important as VTA glutamate neuron activation leads to changes in reward-related responses (Wang et al., 2015; Yoo et al., 2016). Interestingly, the D2 receptor has been detected in VTA glutamate neurons, and the application of D2 agonists is able to hyperpolarize these neurons (Hnasko et al., 2012), suggesting a reciprocal connection between VTA DA and glutamate neurons. VTA glutamate neurons also receive local inhibitory input from GABA neurons, thereby allowing GABA neurons to restrain neuronal activities of VTA glutamate neurons (Yu et al., 2019). Although none has been reported, it is conceivable that VTA glutamate neurons send local excitatory inputs to VTA GABA neurons.

### Long-Range Connections

Given the known heterogeneity of VTA neurons, it is important to investigate the differential remote connectivity of these neurons. VTA DA neurons mainly form synaptic contacts with limbic and cortical regions, including the NACc, amygdala, and PFC (Hnasko et al., 2012; Taylor et al., 2014; Morales and Margolis, 2017). Ventral tegmental area glutamate neurons establish long-range connections either in parallel with or distinct from those of VTA DA neurons. An input–output map has been drawn using a monosynaptic viral tracing approach. In general, VTA glutamate neurons have a grossly similar input–output pattern to VTA DA neurons but with some distinct features (Beier et al., 2015, 2019). For example, similar to VTA DA neurons, VTA glutamate neurons that project to the NACc receive more inputs from the striatal site and fewer projections from the DRN. Yet, distinct from VTA DA neurons, glutamate neurons that project to the PFC and the amygdala receive more inputs from the VP and the LH (Beier et al., 2019).

A) Afferent inputs

Monosynaptic rabies virus tracing results show that VTA glutamate neurons receive strong upstream projections from the LH, DRN, cortical regions, VP, LDTg, and PAG (Faget et al., 2016). Similar to VTA DA neurons, VTA glutamate neurons receive heavy inputs from the LH and the DRN. However, VTA glutamate neurons receive proportionally more projections from cortical regions, while DA neurons receive more projections from striatal regions (Faget et al., 2016). Cortical inputs to VTA glutamate neurons are from a variety of subregions, mainly the somatosensory motor, insular, and cingulate cortices (Faget et al., 2016). Although the general afferent projections to VTA glutamate neurons are clear, specific upstream neuronal subtypes and their detailed mechanisms in the regulation of VTA glutamate neuron activities are largely underexplored.

B) Efferent projections

**FIGURE 1:**
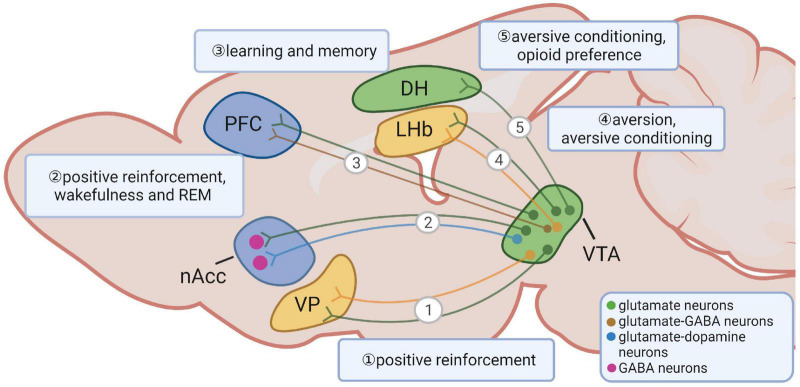
Long-range projections of ventral tegmental area (VTA) glutamate neurons and their functions. VTA glutamate neurons project to the ventral pallidum, the nucleus accumbens, the prefrontal cortex, the lateral habenula and the dorsal hippocampus. The shapes and locations can only represent the general structure and location. The segregated lines with arrows represent a simplified diagram of general projection patterns rather than indicating an existence of parallel and non-overlapping projection patterns from the indicated neurons.

The VTA glutamate neurons send projections that are parallel with VTA DA neurons, such as NACc and PFC, as well as to regions with few VTA dopaminergic inputs, such as LHb and VP (Hnasko et al., 2012; Morales and Root, 2014). As mentioned above, VTA glutamate neurons send abundant projections to the ventral striatum, especially the medial shell of the NACc (Figure 1) (Yamaguchi et al., 2011; Hnasko et al., 2012). TH is present in the majority of VTA glutamate neurons that project to the NACc, and more than half of TH expressing neurons projecting to the NACc co-release glutamate (Stuber et al., 2010; Tecuapetla et al., 2010; Yamaguchi et al., 2011; Hnasko et al., 2012; Mongia et al., 2019), VTA glutamate neurons can drive positive reinforcement by releasing glutamate in the NACc even in the absence of DA release (Zell et al., 2020). VTA glutamatergic projections in the NACc are also sufficient to promote wakefulness and rapid eye movement (REM) independent of DA release (Yu et al., 2019).

Electrophysiological data suggest that glutamate release from the VTA depolarizes PFC neurons and evokes monosynaptic excitatory postsynaptic potentials (EPSPs) (Lavin et al., 2005; Yamaguchi et al., 2011; Gorelova et al., 2012; Hnasko et al., 2012). In addition, VTA glutamate neuron-induced EPSPs in the PFC can be eliminated by glutamate but not DA antagonists (Hnasko et al., 2012). Ultrastructure studies show that ∼60% of PFC projecting VTA neurons contain Vglut2, half of which are TH positive (Yamaguchi et al., 2011; Gorelova et al., 2012), suggesting that Vglut2 positive neurons compose a large portion of mesocortical neurons.

As previously mentioned, VTA glutamate neurons also send abundant projections to structures that receive less DA inputs, such as the LHb and the VP (Figure 1) (Hnasko et al., 2012; Root et al., 2014; Taylor et al., 2014; Yoo et al., 2016). The majority of Vglut2 positive fibers in the LHb lack TH, and DA release is not detected after optical activation of TH^+^ fibers from the VTA (Hnasko et al., 2012; Taylor et al., 2014). Surprisingly, axons from most mesohabenular neurons are found to co-express Vglut2 and markers of GABAergic neurons, and form both symmetric and asymmetric synapses onto LHb neurons (Root et al., 2014, 2018b; Yoo et al., 2016). Selective *ex vivo* activation of mesohabenular axons evokes GABA_A_-mediated outward and AMPA-mediated inward currents on individual neurons (Root et al., 2014). While these neurons co-release two neurotransmitters, glutamate and GABA are packaged and released from distinct pools of vesicles and synapses, suggesting an independent mechanism in accumulation, release, and recycling of each neurotransmitter (Root et al., 2018b). Interestingly, *ex vivo* train activation of glutamatergic projections to the LHb produces a net inhibitory effect, leading to a persistent decrease in the firing rate of postsynaptic cells (Yoo et al., 2016), suggesting a dominant role of GABA over glutamate release.

The VTA Vglut2-expressing fibers are present at the rostra-caudal “finger-like” extent of the VP. Similar to VTA Vglut2-expressing fibers in the LHb, the majority of these fibers lack TH expression (Hnasko et al., 2012). Optogenetic stimulation of these fibers elicits both AMPAR- and NMDAR-mediated currents, suggesting the existence of functional excitatory glutamatergic terminals (Hnasko et al., 2012; Yoo et al., 2016). Interestingly, optical activation induces gabazine-sensitive inhibitory postsynaptic currents (IPSCs) in the VP (Yoo et al., 2016), suggesting that VTA Vglut2 neurons projecting to the VP also co-release GABA. Unlike the net inhibitory effect from activating VTA glutamatergic fibers in the LHb, activation of VTA Vglut2 expressing terminals in the VP elicits a consistent increase of firing in postsynaptic neurons, suggesting that, contrary to what has been found in the LHb, the effect by glutamate release from VTA glutamatergic fibers in the VP dominates over the effect by GABA release (Yoo et al., 2016).

The VTA dopaminergic inputs to the dorsal hippocampus (DH) have long been proposed to be involved in memory modulation (Lisman and Grace, 2005; Mcnamara et al., 2014; Rosen et al., 2015). However, this idea has been challenged due to the scarcity of dopaminergic projections found in the DH (Kempadoo et al., 2016; Takeuchi et al., 2016). A recent study shows that VTA glutamate neurons send one-way projections to the DH (Ntamati and Luscher, 2016; Adeniyi et al., 2020; Han et al., 2020). In addition, these DH-projecting glutamate neurons are TH negative and are mostly located in rostral VTA (Han et al., 2020). Interestingly, postsynaptic currents in the granule cell layer of the dentate gyrus elicited by monosynaptic VTA projections are sensitive to both GABA and AMPA receptor antagonists, suggesting that the upstream VTA neurons co-release GABA and glutamate (Ntamati and Luscher, 2016). Thus, VTA glutamate neurons share parallel projections to those of VTA DA neurons but also exhibit distinct projections that may function independently of DA action.

## Behavioral Regulation by Ventral Tegmental Area Glutamate Neurons

### Behaviors Associated With Changes in Valence

Extensive research has been focused on the functional roles of VTA DA neurons, and these neurons are known to play distinct roles in both positive and negative reinforcement, resulting in preference and avoidance behaviors, respectively (Adcock et al., 2006; Brischoux et al., 2009; Bromberg-Martin et al., 2010). VTA DA neurons respond with increased activities to both rewarding and aversive stimuli (Salamone and Correa, 2012), suggesting physiological implications of these neurons in response to diverse and even conflicting environmental settings. Despite complicated DA neuron responses to seemingly conflicting cues, acute activation of these neurons leads to positive reinforcement and behavioral preference (Tsai et al., 2009; Adamantidis et al., 2011; Witten et al., 2011).

Compared to VTA DA neurons, the role of VTA glutamate neurons seems to be more complicated with inconsistent responses toward rewarding and aversive cues and may induce both preference and avoidance behaviors (Root et al., 2018a). *In vivo* Ca2 + recording data reveal that VTA glutamate neurons exhibit increased overall firing to both aversive and rewarding stimuli (Montardy et al., 2019; Mcgovern et al., 2021). Interestingly, a small population of VTA glutamate neurons increases firing to both aversive and rewarding stimulus (Root et al., 2018a), which might be explained by the functional heterogeneity of different VTA glutamate neuron populations. For instance, an *in vivo* Ca2 + recording study reveals that Vglut2^+^ Vgat^–^ neurons signal cues predicting reward and Vglut2^+^ Vgat^+^ neurons signal unconditioned rewarding and aversive outcomes (Root et al., 2020). However, *in vivo* electrophysiology recording shows that the majority of VTA glutamate neurons decrease their firing, while a small subset shows no change in firing, in response to reward stimulus (Root et al., 2018a,^2020^). This inconsistency might be due to the insensitivity of Ca2 + indicator toward the decrease of neuronal activity (Chen et al., 2013). Nevertheless, these observations implicate VTA glutamate neurons functioning in physiological responses to various environmental cues.

Despite responses of VTA glutamate neurons to both rewarding and aversive cues, direct optical activation of VTA glutamate neurons induces conditioned place preference and appetitive instrumental conditioning (Wang et al., 2015; Yoo et al., 2016). The rewarding effects of VTA glutamate neurons are suggested to be mediated through the activation of VTA DA neurons that project to the NACc (Wang et al., 2015). However, the anatomical connection between VTA glutamate and DA neurons in mediating the rewarding effects remains to be verified. Interestingly, VTA glutamate neurons could induce reinforcement in the absence of DA release (Zell et al., 2020), suggesting a DA-independent effect on rewarding behaviors. However, sustained stimulation of VTA glutamate neurons is less preferred and could even manifest as apparent behavioral avoidance (Yoo et al., 2016). Similarly, in contrast to the previous discussion on inducing rewarding effects, optical stimulation of VTA glutamate neurons may induce aversive escape behaviors (Barbano et al., 2020). The reasons underlying these contrasting observations are unknown but may involve different subsets of VTA glutamate neurons or different stimulation protocols, which may cause different and sometimes opposite effects on neuron activity (Wang et al., 2015; Yoo et al., 2016; Root et al., 2020; Zell et al., 2020). Therefore, VTA glutamate neurons signal both rewarding and aversive stimuli and may induce rewarding or aversive effects.

Reminiscent of conflicting behavioral outcomes by activating VTA glutamate neurons in remote projecting sites, acute activation of VTA long-range glutamatergic terminals also produces diverse and conflicting behaviors. The mesolimbic pathway is important for aversive conditioning (Pezze and Feldon, 2004; Zweifel et al., 2011). Optogenetic activation of VTA Vglut2→NACc projections induces aversion through glutamate-mediated action on local GABAergic interneurons (Qi et al., 2016). The LHb is a brain region known for its function in conditioning aversion and reward (Lammel et al., 2012; Stopper and Floresco, 2014). Acute optogenetic activation of VTA glutamatergic fibers in the LHb elicits aversion and produces aversive conditioning (Root et al., 2014). Acute activation of VTA glutamatergic terminals in the LHb, VP, and NACc induces self-stimulation (Yoo et al., 2016; Zell et al., 2020). VTA glutamatergic projections to the DH are significant for the formation and retrieval of context memories with aversive stimulus, suggesting that VTA Vglut2→DH projections represent negative valence (Han et al., 2020). The reason underlying these apparent discrepancies within VTA glutamatergic projections to different downstream regions is unknown due to the distinct roles of different subsets of VTA glutamate neurons.

### Drug Addiction

The μ-opioid receptors (MORs) are well-known to mediate the rewarding analgesic effects of commonly prescribed and abused opioids (Kieffer and Gaveriaux-Ruff, 2002). It is well established that MORs modulate opioid reward through inhibition of GABA transmission and subsequent DA neuron disinhibition (Steffensen et al., 2006; Hjelmstad et al., 2013; Matsui et al., 2014; Fields and Margolis, 2015). Recent studies suggest that MORs also inhibit glutamatergic transmission onto general Vglut2 expressing synapses, suggesting a role of glutamate neurons in opioid effects (Reeves et al., 2021). In addition, opioid administration brings an immediate effect of increasing cerebral blood flow in the anterior cingulate cortex, thalamus, and amygdala (Schlaepfer et al., 1998; Kosel et al., 2008), followed by long-lasting euphoric effects (Denier et al., 2013), further indicating that glutamate transmission plays a potential role in the “rush” after opioid administration. Histological data reveal the presence of functional MORs in VTA glutamate neurons (Miranda-Barrientos et al., 2021). Opioids, such as morphine, have been shown to activate VTA DA neurons by inhibiting GABAergic inputs and strengthening glutamatergic inputs (Jalabert et al., 2011; Chen et al., 2015). VTA glutamate transmission also plays an important role in reinstatement of heroin seeking (Bossert et al., 2004). Moreover, optically activating the VTA Vglut2→DH projections promotes opioid preference (Han et al., 2020). Thus, increasing evidence suggests that VTA glutamate neurons mediate opioid effects.

While its use in the United States gains more popularity due to the ongoing legalization process (Schulden et al., 2009), cannabis can also be aversive to certain groups of people (D’souza et al., 2004; Parsons and Hurd, 2015). Similar paradoxical effects have also been observed in experimental animals. Δ^9^-Tetrahydrocannabinol (Δ^9^-THC), the primary psychoactive ingredient of cannabis, is rewarding to squirrel monkeys but not to rhesus monkeys (Mansbach et al., 1994; John et al., 2018). In addition, low doses of Δ^9^-THC facilitate intracranial self-stimulation (ISS), while high doses inhibit ISS (Vlachou et al., 2007; Wiebelhaus et al., 2015). The Δ^9^-THC cannabis reward action is believed to be mediated by activation of the CB1 receptor on VTA GABA neurons and disinhibition of VTA DA neurons (Lupica and Riegel, 2005). Yet, the mechanisms underlying the aversive effects of cannabis are unclear. Mounting evidence demonstrates abundant expression of the CB1 receptor in VTA glutamate neurons (Melis et al., 2000, 2004; Han et al., 2017). It is proposed that the aversive effects of cannabis are due to its dose-dependent inhibition of VTA glutamate neurons. Given its rewarding effects through VTA GABA neurons, the overall effects of cannabis depend on the ratio of CB1 expression levels in the VTA GABA over VTA glutamate neurons (Han et al., 2017).

The VTA is also an important site mediating the nicotine effects. Nicotine addiction is mediated through the nicotinic acetylcholine receptor (nAChR). The expression of β2-containing nAChRs has been detected in VTA DA neurons (Klink et al., 2001; Changeux, 2010), and the nicotine treatment promotes VTA DA release (Mansvelder and Mcgehee, 2002). In addition, blocking nicotine signaling in the VTA prevents the induction of nicotine preference (Tolu et al., 2013; Durand-De Cuttoli et al., 2018). Recently, nicotinic receptors have been found in VTA glutamate neurons (Yan et al., 2018), and application of nicotine induces glutamatergic excitatory potentiation on VTA DA neurons (Gao et al., 2010; Mao et al., 2011; Yan et al., 2018). In addition, VTA glutamatergic signaling has also been implicated in regulating the reinstatement of cocaine seeking (You et al., 2007; Wise and Morales, 2010; Williams et al., 2014) and benzodiazepine effects (Heikkinen et al., 2009) by regulating VTA DA signaling. These data suggest that VTA glutamatergic inputs to DA neurons might play a role in nicotine addiction.

### Other Behaviors

A) Learning and memory

The VTA glutamate neurons are known to form asymmetrical synapses and evoke EPSCs on the PFC and the DH, both of which are known to regulate learning and memory (Miller and Cohen, 2001; Curtis and D’esposito, 2003; Matus-Amat et al., 2004). Within the PFC, VTA glutamatergic projections function as a signal for rapid behavioral responses, such as prediction error signaling (Gorelova et al., 2012). Within the DH, VTA glutamatergic projections signal negative valence to memory circuits, leading to the formation of fear-inducing context memories and to context-specific reinstatement of fear (Han et al., 2020). Thus, VTA glutamate neurons play a role in memory consolidation, at least through projecting to PFC and DH.

B) Sleep and wakefulness

The VTA DA neurons are known to promote wakefulness through DA D2-like receptors (Eban-Rothschild et al., 2016; Oishi et al., 2017). VTA glutamate neurons increase activity during wakefulness and REM sleep while decreasing firing during non-REM sleep (Yu et al., 2019). Chemogenetic activation of VTA glutamate neurons increases the duration of wakefulness during the inactive phase, and ablation of VTA glutamate neurons reduces wakefulness and increases NREM sleep during the active phase (Yu et al., 2019). It will be interesting to understand the functional anatomy of VTA glutamate neurons with the known brain circuits regulating the sleep-wake cycle.

C) Defensive behaviors

The VTA glutamate neurons encode danger signals and are required for the innate escape behavior in response to threatening stimuli (Barbano et al., 2020). Lateral hypothalamus glutamate neurons are known to increase the activity to predator odors, and optogenetic activation of LH glutamate neurons induces innate defensive behaviors (Lecca et al., 2017; Mangieri et al., 2018; Chen et al., 2020). Ultrastructure and electrophysiology data show that LH glutamate neurons send projections to VTA glutamate neurons (Kempadoo et al., 2013; Barbano et al., 2020). Interestingly, LH glutamate neurons send projections to the paraventricular nucleus (PVH) glutamate neurons, which elicit strong escape jumping behaviors upon activation (Mangieri et al., 2018). PVH glutamate neurons can in turn activate putative VTA glutamate neurons for defensive behaviors (Mangieri et al., 2019). Thus, LH glutamate neurons promote defensive behaviors through both direct projections and indirect projections *via* PVH glutamate neurons to VTA glutamate neurons.

D) Eating behaviors

The VTA neuronal signaling has been found to be negatively correlated with obesity development in human (Baik, 2013; Guo et al., 2014), and specifically, suggesting that patients with obesity have a tendency to consume more calories for compensating the low level of DA signaling. In rodents, VTA neurons have been demonstrated to mediate the function of energy balance-related hormones in feeding and body weight regulation (Fulton et al., 2006; Hommel et al., 2006; Narayanan et al., 2010; Perello and Zigman, 2012; Cone et al., 2014). Although most studies focus on VTA DA neurons, the functional roles of VTA glutamate neurons have been emerging. Especially, activation of glucagon-like peptide-1 (GLP-1) neurons in the VTA inhibits feeding by increasing AMPA/Kainate signaling in VTA glutamate neuron projections to the NACc (Mietlicki-Baase et al., 2014), suggesting the role of VTA glutamate neurons in regulating the mediation of GLP-1 from the VTA in feeding behaviors. In addition, the LH and PVH are two important regions for feeding behaviors and body weight homeostasis (Gutierrez et al., 2011; Bonnavion et al., 2016; Krashes et al., 2016; Sutton et al., 2016). Since these two regions send glutamatergic projections or GABAergic projections to VTA glutamate neurons or GABA neurons that regulate VTA glutamate release (Nieh et al., 2016; Tyree and De Lecea, 2017; Mangieri et al., 2019; Barbano et al., 2020), VTA glutamate neurons are well-positioned to modulate homeostatic feeding and body weight balance. Given the known role of VTA in mediating hedonic feeding and VTA glutamate neurons in regulating positive reinforcement (Rossi and Stuber, 2018), these neurons might also be involved in hedonic feeding and contribute to diet-induced obesity. Future studies are warranted to address the function of these neurons in homeostatic and hedonic feeding as well as body weight regulation.

## Author Contributions

JC wrote the manuscript with significant inputs from QT. Both authors contributed to the article and approved the submitted version.

## Conflict of Interest

The authors declare that the research was conducted in the absence of any commercial or financial relationships that could be construed as a potential conflict of interest.

## Publisher’s Note

All claims expressed in this article are solely those of the authors and do not necessarily represent those of their affiliated organizations, or those of the publisher, the editors and the reviewers. Any product that may be evaluated in this article, or claim that may be made by its manufacturer, is not guaranteed or endorsed by the publisher.
